# Effect of Fiber Fraction on Ballistic Impact Behavior of 3D Woven Composites

**DOI:** 10.3390/polym15051170

**Published:** 2023-02-25

**Authors:** Xiaoping Shi, Ying Sun, Jing Xu, Li Chen, Ce Zhang, Guoli Zhang

**Affiliations:** 1 School of Textile Science and Engineering, Tiangong University, Tianjin 300387, China; 2 Ministry of Education Key Laboratory of Advanced Textile Composite Materials, Institute of Composite Materials, Tiangong University, Tianjin 300387, China; 3 AVIC Aerospace Life-Support Industries LTD, Xiangyang 441003, China

**Keywords:** 3D woven composites, fiber volume fraction, ballistic impact behavior, ballistic limit velocity, specific energy absorption, para-aramid fiber

## Abstract

This paper studies the ballistic impact performance of 3D woven composites (3DWCs) with hexagonal binding patterns. Para-aramid/polyurethane (PU) 3DWCs with three kinds of fiber volume fraction (V_f_) were prepared by compression resin transfer molding (CRTM). The effect of V_f_ on the ballistic impact behavior of the 3DWCs was analyzed by characterizing the ballistic limit velocity (V50), the specific energy absorption (SEA), the energy absorption per thickness (E_h_), the damage morphology and the damage area. 1.1 g fragment-simulating projectiles (FSPs) were used in the V50 tests. Based on the results, when the V_f_ increases from 63.4% to 76.2%, the V50, the SEA and the E_h_ increase by 3.5%, 18.5% and 28.8%, respectively. There are significant differences in damage morphology and damage area between partial penetration (PP) cases and complete penetration (CP) cases. In the PP cases, the back-face resin damage areas of the sample III composites were significantly increased to 213.4% of the sample I counterparts. The findings provide valuable information for the design of ballistic protection 3DWCs.

## 1. Introduction

In the last thirty years, para-aramid-fiber-reinforced polymer composites have been developed for ballistic protection applications due to their advantages of light weight, high strength, high modulus, high toughness and excellent energy absorption performance [1,2,3,4,5,6,7,8,9]. The dominant fiber reinforcements have a unidirectional prepreg or a 2D woven laminated structure [10,11,12,13]. Various types of 3D woven composites (3DWCs) have been presented to overcome the shortcomings of laminates. The potential benefits of 3D woven reinforcements include reduced fabrication cost, improved impact resistance, greater design flexibility and superior though-thickness mechanical properties. These 3DWCs have impressive potential applications in bullet-proof vests, ballistic helmets, and other products related to high-velocity impact and military uses [14,15,16].

Min and Chen [17] investigated the effect of ply areal density on the ballistic performance of Twaron/Epoxy 3DWCs with a total areal density (AD) of 12 kg/m^2^ and a fiber volume fraction (V_f_) of 55%. The findings indicated that reinforcements with larger ply areal density and a smaller number of plies suffered less ballistic damage. Chen [18] studied the ballistic impact energy absorption and damage morphology of 3DWCs using perforated ballistic tests and X-ray CT. The tests revealed that the 3DWCs exhibit a 3.3% energy absorption increase and a 65.3% delamination damage volume decrease compared to 2D plain composites at an equivalent AD. The mechanical and failure behavior under ballistic impact of Kevlar/carbon hybrid 3DWCs was investigated by Zheng [19]. The V_f_ values of the composites were from 52.3% to 57.25%. The results showed that the damage to the Kevlar layer was due to fiber fracture and pull-out, while in carbon layers, damage was caused by matrix cracking and brittle-fiber failure. Ha-Minh [20] studied the ballistic impact behavior of 3D woven preforms with an areal density of 1.66 kg/m^2^. In the fabric plane, the density of the warp yarns was 20 ends/cm and that of the weft yarns was 27.8 picks/cm. Fragment-simulating projectiles (FSPs) with a diameter of 5.45 mm and a weight of 1.11 g were used for the ballistic tests. The results showed that partial penetration (PP) occurred at an impact velocity of 306m/s and complete penetration (CP) occurred at an impact velocity of 400 m/s. Abtew [21,22] compared the ballistic performance of 2D plain fabric and 3D woven fabric panels according to NIJ standard-0101.06 Level-IIIA. The 3D woven fabric comprised 5-layer warp yarns and 6-layer weft yarns. All yarns were para-aramid (Twaron^®^ 930dTex, Teijin Aramid, a subsidiary of Teijin Group, The Netherland.) with an areal density of 1 kg/m^2^. In the fabric plane, the total yarn density was 52.5 ends/cm for warp yarns and 52.5 picks/cm for weft yarns. Based on the results, 8 kg/m^2^ of 2D plain weave and corresponding 3D warp interlock fabric panels showed similar deformation and energy absorption capability. However, 6 kg/m^2^ of 2D plain fabric still showed good ballistic performance compared to its counterpart 3D warp interlock fabric [23]. The excellent mouldability enables the 3D woven flat preform to form a curved surface structure through the stamping method for items such as 3D woven female body armor and bulletproof helmets [24]. It is difficult to improve ballistic protective performance while maintaining moldability. To satisfy these two contradictory goals, Chen and Yang [25] compounded through-the-thickness angle-interlock and layer-to-layer orthogonal-interlock structures. The results showed that the ballistic performance of the compound preform was better than the through-the-thickness angle-interlock, and equivalent moldability could also be provided. Bandaru [26] studied the effect of the stacking method on the ballistic properties of Kevlar/polypropylene composites. The results showed that symmetrical stacking exhibited better ballistic penetration resistance, successfully resisting the impact of 9 mm full metal jacket (FMJ) projectiles with a velocity of 365–395 m/s. A large number of numerical studies have been carried out on 3D woven preforms and composites due to the complexity of the ballistic penetration process and the difficulty of capturing the internal damage phenomena [27,28,29,30,31,32,33,34,35,36,37,38,39,40]. Gu and Sun [41,42] reported the ballistic impact damage to 3DWCs under penetration by a hemispherical rigid projectile using a multi-scale geometrical model of the 3DWCs and high-strain-rate constitutive equations for fibertows. V_f_ is a key parameter affecting the mechanical properties of fiber-reinforced composites. In the ballistic performance literature discussed above, the V_f_ of 3DWCs is often below 60% [43,44,45,46,47], but the V_f_ of UD/2D laminates for ballistic protection applications can reach 80% or more. The mechanism by which V_f_ affects the ballistic impact behavior of 3DWCs is still unclear and needs further study. In order to determine the relationship between ballistic performance and V_f_, it is necessary to increase the V_f_ of 3DWCs, which is why the present study is necessary.

Combined with innovative reinforcement structure, para-aramid fabrics may provide more effective ballistic composites. In this paper, a 3D woven architecture with a hexagonal bonding pattern was designed and three kinds of 3DWCs with different V_f_ (up to 76.2%) were manufactured using a CRTM process. The effects of V_f_ on ballistic impact behavior of 3D woven para-aramid/PU composites against 1.1 g FSPs were investigated experimentally. In order to reveal the effects of V_f_ on the ballistic penetration resistance of the 3DWCs, the damage mechanisms of the 3DWCs were described by characterizing V50, SEA, E_h_, damage morphology and damage area. These results provide further understanding and basis for the design of lightweight and efficient bulletproof 3D woven composite structures.

## 2. Materials and Testing Methods

### 2.1. Materials

#### 2.1.1. 3D Woven Architecture

**Figure 1:**
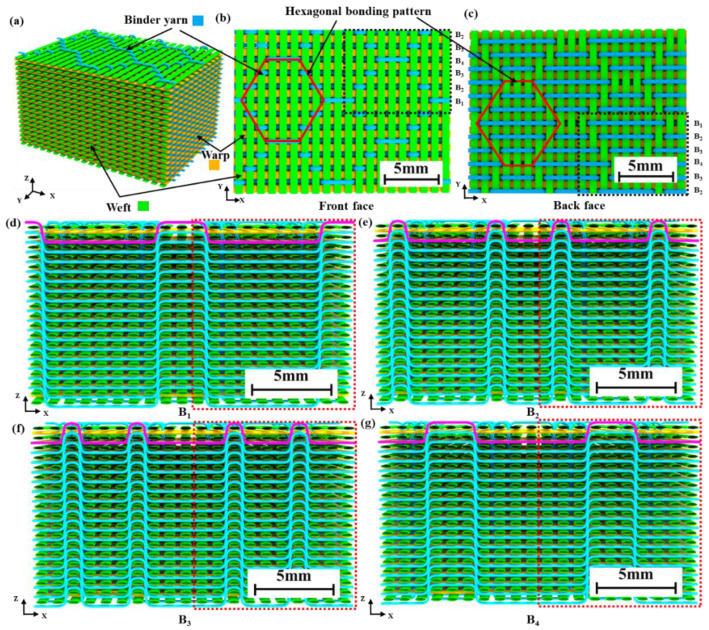
3D woven architecture with hexagonal bonding pattern: (**a**) isometric drawing; (**b**) front face; (**c**) back face; (**d**) binder yarn B_1_; (**e**) binder yarn B_2_; (**f**) binder yarn B_3_; (**g**) binder yarn B_4_.

**Figure 2:**
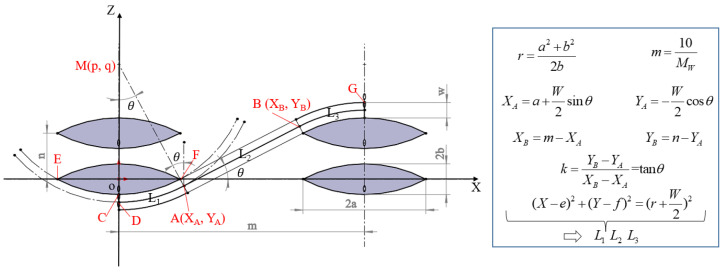
Method for calculating length of curved segment of binder yarn [48].

As shown in Figure 1a, a 3D woven architecture with a hexagonal bonding pattern was designed. This architecture consisted of 18 layers of warp yarns, 18 layers of binder yarns and 19 layers of weft yarns. The linear density of the warp yarns and weft yarns was the same, and was twice that of the binder yarns. The warp yarns and binder yarns were arranged in a 1:1 ratio. The front- and back-face weave patterns are shown in Figure 1b,c. The front face was mainly weft yarn floats, while the back face was mainly binder yarn floats. In this paper, the X, Y and Z directions represent the warp, weft and thickness directions of the 3D woven preform, respectively. The dashed black box is a representative unit along the X-Y plane. This architecture included four types of binder yarn, namely, B_1_, B_2_, B_3_ and B_4_, as shown in Figure 1d–g. The dashed red box is a representative unit along the X–Z plane. The V_f_ of the 3D woven architecture can be calculated according to the yarns of the three systems. Both warp and weft yarns were straight. The length of the curved segment of binder yarn can be calculated as shown in Figure 2.

#### 2.1.2. Specimen Preparation

Tcparan^®^ para-aramid yarns (Yantai Tayho Advanced Materials Co., Ltd., Yantai, China) (1110dtex) were selected to prepare the 3D woven preforms (3DWPs). In this paper, the 3DWPs were produced using a weaving loom developed at the Institute of Composite Materials of Tiangong University. According to ASTM D1777, the thickness (h) of a 3DWP is 12.4 mm. The specifications of the 3DWPs are shown in Table 1.

**Figure 3:**
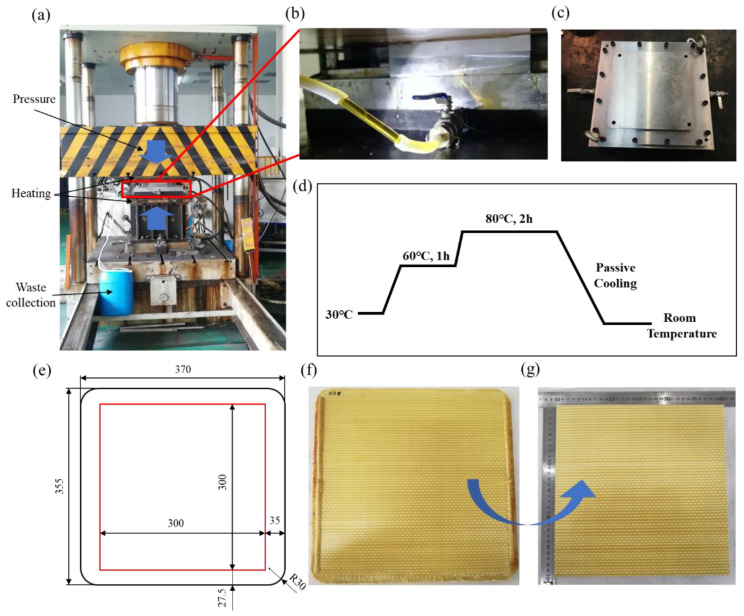
Details of specimen preparation: (**a**–**c**) pressure equipment and mold; (**d**) curing process of two-component polyurethane resin system; (**e**) cutting scheme; (**f**) manufactured sample; (**g**) cut sample.

A two-component polyurethane resin system (PU) consisting of polyol and polymethylene polyphenylene isocyanate (mixed at a volume ratio of 100:73) was used. The mechanical properties of the Tcparan^®^ yarns and PU are listed in Table 2. All 3DWCs were prepared using the compression resin transfer molding (CRTM) method. The details of specimen preparation are shown in Figure 3. The cavity size of the mold was 355 mm × 370 mm, and the radius of the four rounded corners was 30 mm. A mold photo is shown in Figure 3c. As shown in Figure 3a,b, the mold needs to be used with pressure equipment, which provides pressure and heating. In order to improve efficiency, it is necessary to create a vacuum of −0.1 MPa before resin injection. During the resin injection process, the compression pressure was set at 4–6 MPa, and the temperature of the resin and mold were maintained at 30 °C. The resin injection process adopted an equal-flow injection method (20 mL/min), which lasted about 20 min. Compression was carried out after the injection was completed. Different compression pressures need to be set for the preparation of different V_f_ composites. In this study, the preparation pressures were 6.5 MPa, 8 MPa and 15 MPa, respectively. The curing process of the PU infused panels is shown in Figure 3d. Figure 3f is a picture of the back face of a manufactured sample. Based on the requirements of GJB 4300A-2012 [49] for sample size, the manufactured sample was cut according to the scheme in Figure 3e, and a ballistic test sample with a size of 300 mm × 300 mm was obtained, as shown in Figure 3g. To study the effect of V_f_ on the ballistic impact behavior of the hexagonal bonding 3DWCs, three kinds of 3DWCs with different V_f_ were manufactured by compacting the preform according to the specifications shown in Table 3. The V_f_ of the 3DWC was obtained according to the weight and dimensions of the 3DWP, calculated using Equation (1):

(1) $$V_{f}=\frac{10^{3}\times M_{f}}{\rho_{f}\times L_{x}\times L_{y}\times H}\times 100\%$$

where $M_{f}$ is the mass of the 3DWP (g); $\rho_{f}$ is the density of the para-aramid fiber (g/cm^3^); and $L_{x}$, $L_{y}$ and H are the length, width and thickness of the 3DWC, respectively (mm).

### 2.2. Ballistic Impact Tests

**Figure 4:**
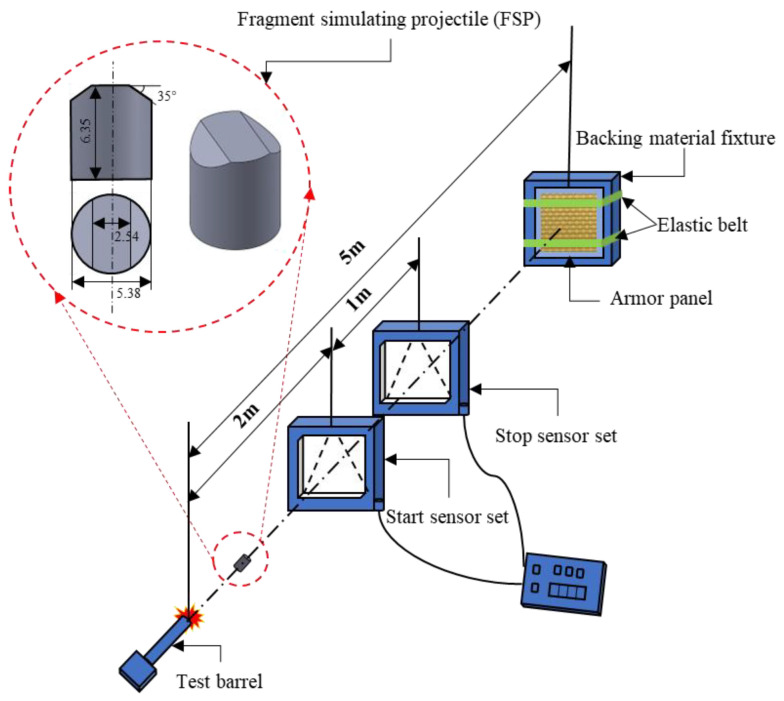
Schematic diagram of ballistic limit V50 test system.

For the ballistic impact tests, the standard test method GJB 4300A-2012 (Requirements of safety technical performance for military body armor) was followed. As shown in Figure 4, the samples were placed 5 m from the tip of the gun muzzle, firmly tied to the backing material fixture. The projectiles are 45-steel 17-grain (1.1 g) 0.22 caliber FSPs, with a diameter of 5.38 mm, a height of 6.35 mm, and a surface hardness of HRC29 ± 2. The projectiles were fired from a special 7.62 mm caliber fragment firing barrel and hit the target at a 90° incidence. All the ballistic tests presented in this paper were performed in the Quality Test Center for Special Protective Clothing of the CPLA (Chinese People’s Liberation Army).

V50 is the velocity at which, using a given projectile and target material, the estimated probability of perforation is 0.5. According to GJB 4300A-2012, V50 is calculated using the following method: an even number (at least six) shots are used for the calculation. Half of the shots must perforate the target material (complete penetration (CP)). Half of the shots must not perforate (partial penetration (PP)) the target material. The highest recorded velocity of the group (half PPs and half CPs) must not be more than 38 m/s higher than the lowest of the group. The measurement is the arithmetic mean of the group of velocities. The impact velocity of the projectile is calculated based on the time it passes through two timers with a constant distance. There is a velocity attenuation of bullet velocity from the timer to sample, so V50 can be obtained by subtracting the attenuation velocity from the velocity measuring point to the target sample.

### 2.3. Characterization of Damage Morphology

A 3D contour measurement instrument (KEYENCE VR-5200, Osaka, Japan) was used to extract the damage area around the impact point on the sample surface, including the damage area and shape of the front and back of the target and the residual deformation height on the back of the target.

## 3. Results and Discussion

### 3.1. Ballistic Test Results

V50 is calculated based on Equations (2)–(4):

(2) $$V50=\bar{V}-\Delta V$$

where $\bar{V}$ is the average velocity of the effective FSP at the speed measurement point (m/s), and ΔV is the velocity attenuation of the FSP from the speed measurement point to the target (m/s).

(3) $$\bar{V}=\sum_{1}^{n}\frac{V_{i}}{n}$$

where $V_{i}$ is the velocity of the i round effective FSP at the speed measurement point (m/s), and n is the number of rounds with effective FSP velocity at the speed measurement point.

(4) $$\Delta V=C_{43}\frac{10X}{\Delta D\left(V\right)}$$

where $C_{43}$ is the ballistic coefficient of the FSP, X is the distance from the speed measurement point to the target (m), and ΔD(V) is the increment of the Siacci function, which can be obtained from the Siacci function table according to the V value.

**Figure 5:**
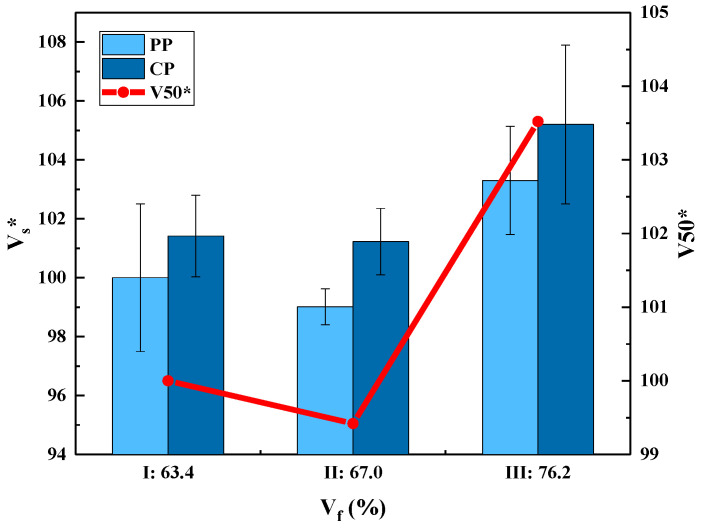
Results of ballistic impact tests, V_s_*-V_f_ and V50*-V_f_. (Note: Both V_s_* and V50* are normalized with the corresponding values of sample I.).

V50 and the effective FSP velocity at the speed measurement point of the three kinds of 3DWCs are shown in Figure 5. It was observed that sample III (V_f_ = 76.2%) showed the best response. However, the V50 values for sample I (V_f_ = 63.4%) and sample II (V_f_ = 67.0%) are almost equal. A concrete conclusion cannot be drawn merely by comparing the V50 values. The areal density (AD) and the thickness are two important parameters of the 3DWCs. Therefore, energy absorption capability was further examined using the specific absorption energy (SEA) and energy absorption per unit thickness (E_h_), which can be calculated using Equations (5)–(7).

### 3.2. Energy Absorption Capability of 3DWCs

The energy absorbed by the V50 test (E_V50_) can be calculated using Equation (5):

(5) $$E_{V50}=\frac{1}{2}m{\left(V50\right)}^{2}$$

where m is the mass of the FSP (kg).

SEA can be computed using Equation (6):

(6) $$SEA=\frac{E_{V50}}{AD}$$

where AD is the areal density of the 3DWC sample (kg/m^2^).

E_h_ can be computed using Equation (7):

(7) $$E_{h}=\frac{E_{V50}}{h}$$

where h is the thickness of the 3DWC sample (mm).

**Figure 6:**
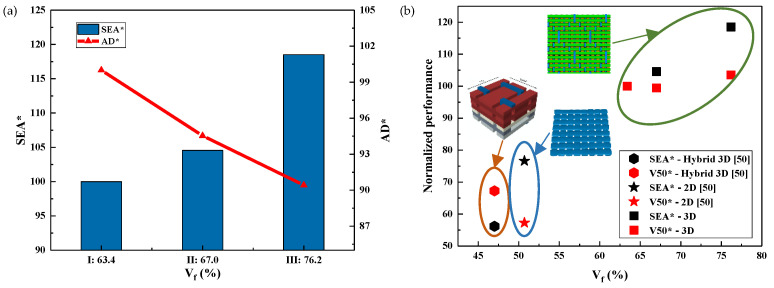
Experimental results of impact tests: (**a**) relationship between normalized performance (SEA* and AD*) and V_f_; (**b**) relationship between normalized performance (V50* and SEA*) and V_f_. (Note: V50*, SEA* and AD* are normalized with the corresponding values of sample I.) [50].

It can be seen from Figure 6 that V_f_ has a significant effect on the V50 and SEA values of fiber composites. Moreover, the V50 and SEA of the 3DWCs with hexagonal binding pattern designed in this study are almost twice that of the hybrid 3DWCs and 2D laminates. Therefore, the 3DWCs with hexagonal binding pattern have obvious advantages in improving the ballistic limit and specific energy absorption of composites.

**Figure 7:**
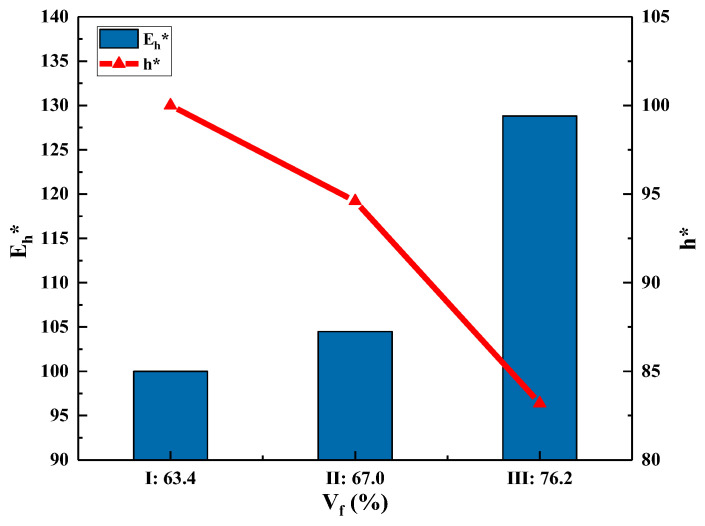
Experimental results of impact tests, E_h_*-V_f_ and h*-V_f_. (Note: E_h_* and h* are normalized with the corresponding values of sample I).

Combining Figure 6a and Figure 7, it can be seen that when V_f_ increases from 63.4% to 76.2% (an increase of 20.2%), both the AD and the thickness decrease significantly, by 9.6% and 17.2%, respectively. Meanwhile, SEA and E_h_ increases by 18.3% and 28.5%, respectively. To sum up, the increase in V_f_ not only helps to reduce the AD and thickness of the 3DWCs, but also significantly improves the energy absorption performance of the 3DWCs. During the design of ballistic protection armor, weight should be taken into as much consideration as ballistic performance. Therefore, it is of great significance to improve V_f_ for the design of lightweight and high-strength bulletproof 3DWCs.

### 3.3. Damage Morphology and Damage Area of 3DWCs

**Figure 8:**
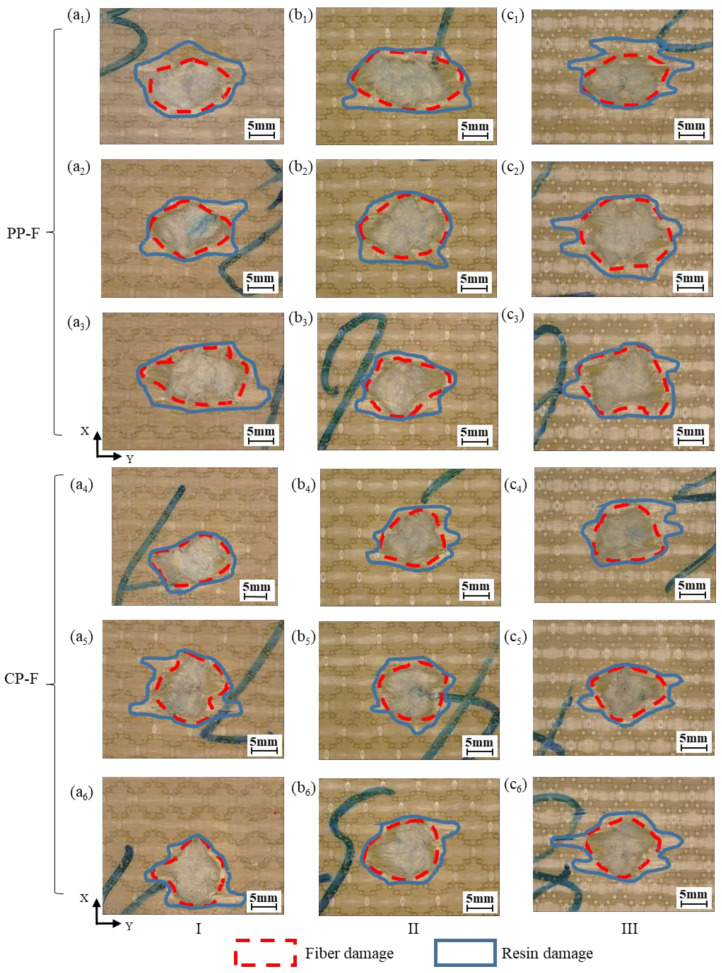
Fiber/resin damage morphology of composite panel after ballistic tests of front face: (**a_1_**)−(**a_3_**) PP-F of sample I; (**a_4_**)−(**a_6_**) CP-F of sample I; (**b_1_**)−(**b_3_**) PP-F of sample II; (**b_4_**)−(**b_6_**) CP-F of sample II; (**c_1_**)−(**c_3_**) PP-F of sample III; (**c_4_**)−(**c_6_**) CP-F of sample III. (PP-F: front face of partially penetrated panel; CP-F: front face of completely penetrated panel.)

**Figure 9:**
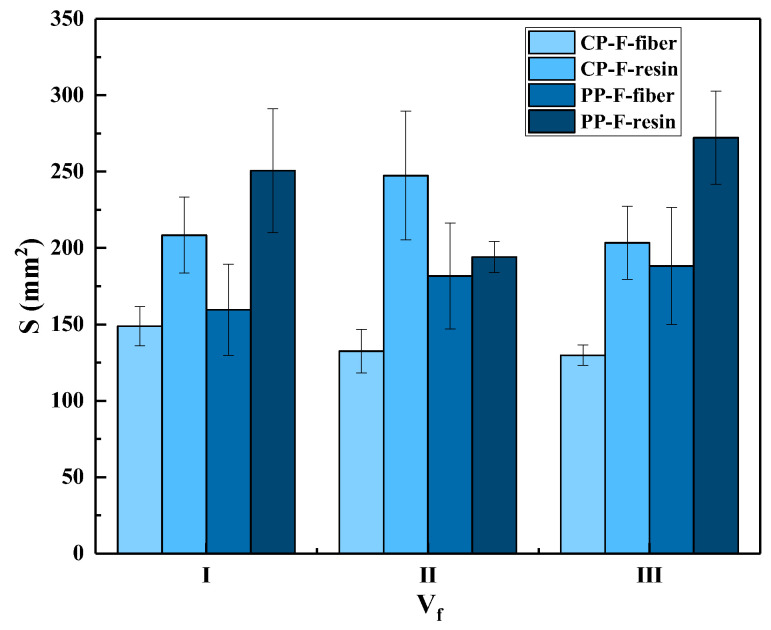
Fiber/resin damage area of composite panel after ballistic test of front face.

It can be seen in Figure 8 that the main failure modes of the impact surfaces of the 3DWCs are fiber breakage and matrix cracking. We statistically analyzed the fiber and resin damage areas; the results are shown in Figure 9. On the front surface, partial penetration (PP) and complete penetration (CP) samples have similarly shaped bullet holes, namely, an ellipse with an approximate long axis along the weft yarn direction (Y-direction). In the PP samples, the fiber damage area is larger than that of the CP samples. This because in the PP samples, more fibers are involved in energy dissipation to capture the FSP, so the area of fiber damage is larger. With the increase in V_f_, the damage area of the PP samples shows an increasing trend. This is because the increase in V_f_ means a decrease in resin volume content (V_r_), which leads to a decrease in constraint on the fiber, resulting in an increase in the fiber damage area.

**Figure 10:**
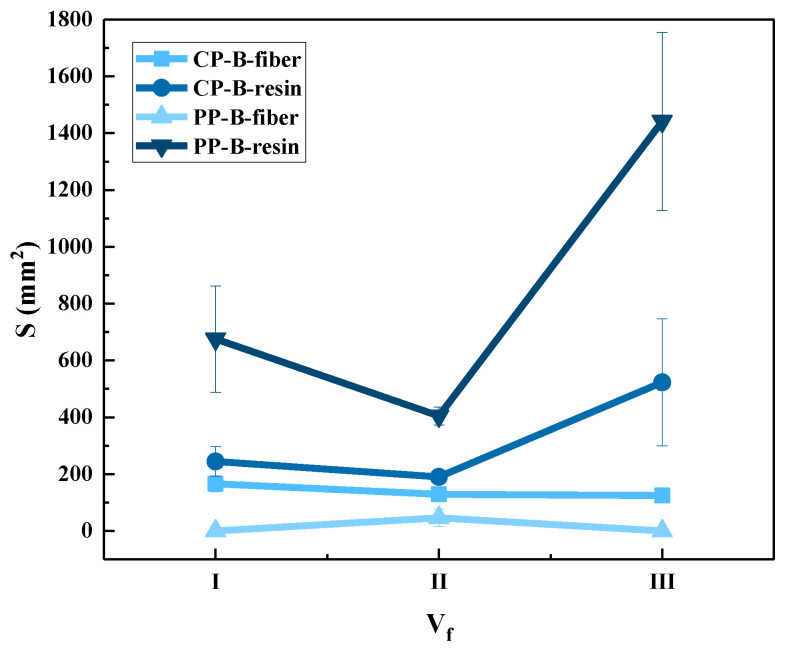
Fiber/resin damage areas of samples after ballistic test of back face.

Statistics for the fiber damage areas and resin damage areas on the back faces of the tested panels are shown in Figure 10. Combined with Figure 8 and Figure 9, the figure shows that the area of fiber damage on the back face is only slightly larger than that on the front. This shows that the energy dissipation mode of the 3DWCs is mainly shearing when CP occurs. In the case of PP, the resin damage area on the back face is much larger than on the front face. This indicates that the failure mode in PP is more complex. In regard to the energy dissipation mechanism, the front face is dominated by fiber and resin shearing, while the back face includes resin cracking, fiber-resin interface damage, fiber tensile deformation and fracture. The resin damage area on the back face in CP is much smaller than that in PP. Therefore, it can be said that the increase in the damage resin area on the back face is helpful to improve the protective performance of 3DWCs. With the increase of V_f_, the resin damage area on the back face of the 3DWCs shows a significant upward trend. Therefore, the increase in V_f_ helps to increase the amount of fibers and resin involved in energy dissipation by increasing the damage area on the back side, thus contributing to the improvement of ballistic protection performance.

**Figure 11:**
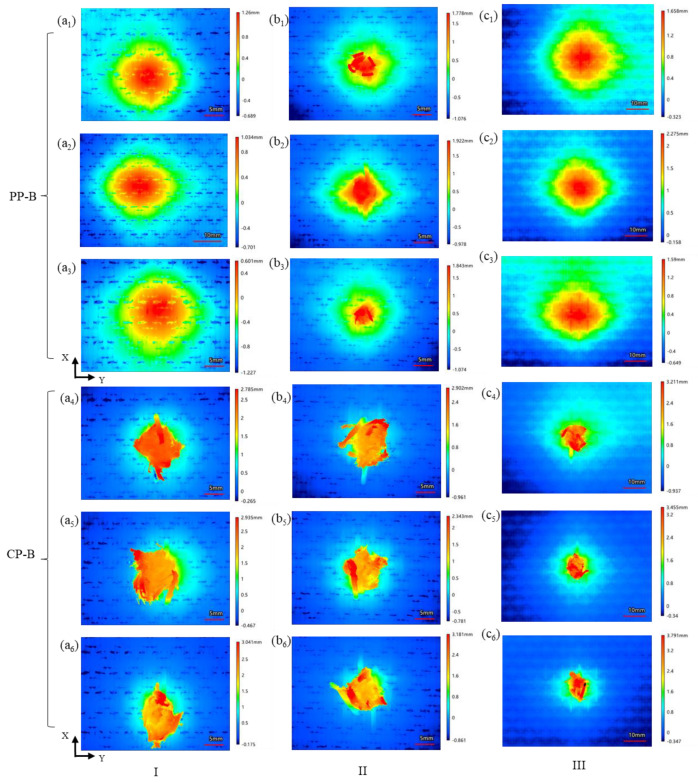
3D profiler scan results for back-face damage morphology of the composite panel samples: (**a_1_**)−(**a_3_**) PP-B of sample I; (**a_4_**)−(**a_6_**) CP-B of sample I; (**b_1_**)−(**b_3_**) PP-B of sample II; (**b_4_**)−(**b_6_**) CP-B of sample II; (**c_1_**)−(**c_3_**) PP-B of sample III; (**c_4_**)−(**c_6_**) CP-B of sample III. (PP-B: back face for partial penetration; CP-B: back face for complete penetration).

Figure 11 shows the height variation of the different regions on the back face of the 3DWCs obtained by 3D contour scanning. There are obvious differences between the PP case and the CP case. The height variation for CP was concentrated in an area with a diameter of 10–20 mm centered on the shooting point. The PP case shows a height cloud map approximating concentric circles. With the increase of V_f_, the range of the height variation area shows an increasing trend. In addition, the near-circular height map shows that the 3DWP structures have similar longitudinal-latitudinal bearing capacity. The 3DWP structure helps the fibers of each component jointly bear and dissipate the kinetic energy of the FSP so as to show a nearly in-plane isotropic ballistic effect.

**Figure 12:**
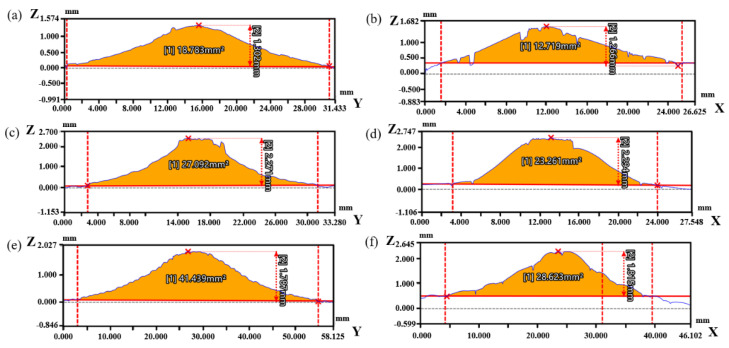
Cross-sectional diagram of representative shooting points in PP: (**a**) Y–Z cross section of sample I; (**b**) X–Z cross section of sample I; (**c**) Y–Z cross section of sample II; (**d**) X–Z cross section of sample II; (**e**) Y–Z cross section of sample III; (**f**) X–Z cross section of sample III.

**Figure 13:**
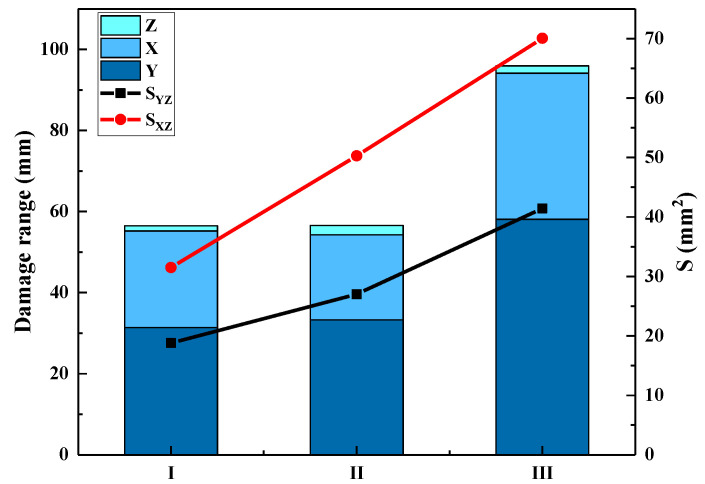
Statistical results for damage range span and damage areas.

Figure 12 shows a cross-sectional diagram of representative shooting points in PP: (a) Y–Z cross section of sample I; (b) X–Z cross section of sample I; (c) Y–Z cross section of sample II; (d) X–Z cross section of sample II; (e) Y–Z cross section of sample III; (f) X–Z cross section of sample III. The X, Y and Z values represent BFS dimensions along the warp, weft and thickness directions, respectively. The Y–Z and X–Z curves are the contours of the BFS along the warp and weft directions. The areas enclosed by the BFS contour curve and coordinate axes are represented by S_YZ_ and S_XZ_, respectively. The statistical results of these parameters are shown in Figure 13. It can be seen that S_YZ_ is always larger than S_XZ_, and the deformation span in the weft direction is always larger than the warp direction. In all three kinds of 3DWCs, when V_f_, X, and Y increase, S_YZ_ and S_XZ_ both increase.

## 4. Conclusions

In this paper, a 3D woven preform with hexagonal binding pattern was designed for the first time. This architecture not only has a high in-plain fiber content, but also has a high degree of orientation due to the low number of interlacings and through-layers of the binding yarns. Para-aramid/PU 3DWCs with three kinds of V_f_ were prepared using the CRTM method. This molding method helped to straighten the 3DWCs fibers and further improved V_f_ to 76.2%. In ballistic tests, the effect of V_f_ on the ballistic performance of the 3DWCs against 1.1 g FSPs was studied. Based on the test results, the following conclusions can be drawn:

1. V_f_ was the key factor affecting the V50 value of the 3DWCs. The ballistic results showed that when V_f_ increased from 63.4% to 76.2%, V50 still increased by 3.5% even when the thickness and AD decreased by 17.2% and 19.6%, respectively.
2. The fiber and resin damage of the impact surface of the 3DWCs occurred in a small area centered on the shooting point, and the damage area was about 1.5~3 times the area of the projectile. With an increase in V_f_, the damage area on the impact surface was slightly increased due to the lower resin content, which weakened the restraint of the resin on the fibers.
3. When V_f_ was 63.4%, stiffness of the 3DWCs was high and deformation occurred only in a small area. When V_f_ was increased to 76.2%, the deformation area significantly increased (by 213.4%), more fiber participated in energy dissipation, and SEA and E_h_ increased by 18.5% and 28.8%, respectively.

The findings of this study provide valuable information for the engineering design and manufacture of 3D woven ballistic composites with higher protection ability. Further studies will be conducted to improve the fiber volume content of 3D woven composites, including more experimental work and numerical modelling.

## Figures and Tables

**Table 1:** Specifications of the 3DWP.

Parameter	Value
Number of warp yarn layers	18
Number of weft yarn layers	19
Number of binder yarn layers	18
Warp yarn density per layer (ends/cm)	8
Weft yarn density per layer (picks/cm)	10
Binder yarn density per layer (ends/cm)	8
Warp yarn linear density (dtex)	1100 × 2
Weft yarn linear density (dtex)	1100 × 2
Binder yarn linear density (dtex)	1100
h (mm)	12.4

**Table 2:** Mechanical properties of Tcparan^®^ yarns and PU matrix.

Materials	Density(g/cm^3^)	Young’s Modulus (GPa)	Tensile Strength (GPa)	Elongation at Break (%)
Tcparan^®^ yarns	1.44	97	3.425	3.75
PU	1.20	3.4	0.085	>5

**Table 3:** Specifications of 3DWCs.

Sample	AD (kg/m^2^)	V_f_ (%)	M_f_ (%)	H (mm)
I	12.5	63.4	72.7	9.9
II	11.8	67.0	76.9	9.3
III	11.3	76.2	80.4	8.2

## Data Availability

The data that support the findings of this study are available within the article.
